# Experimental Comparison and Evaluation of the Affymetrix Exon and U133Plus2 GeneChip Arrays

**DOI:** 10.1371/journal.pone.0000913

**Published:** 2007-09-19

**Authors:** Diana Abdueva, Michele R. Wing, Betty Schaub, Timothy J. Triche

**Affiliations:** Department of Pathology and Laboratory Medicine, Children's Hospital Los Angeles, Keck School of Medicine, University of Southern California, Los Angeles, California, United States of America; Texas A&M University, United States of America

## Abstract

**Background:**

Affymetrix exon arrays offer scientists the only solution for exon-level expression profiling at the whole-genome scale on a single array. These arrays feature a new chip design with no mismatch probes and a radically new random primed protocol to generate sense DNA targets along the entire length of the transcript. In addition to these changes, a limited number of validating experiments and virtually no experimental data to rigorously address the comparability of all-exon arrays with conventional 3′-arrays result in a natural reluctance to replace conventional expression arrays with the new all-exon platform.

**Methodology:**

Using commercially available Affymetrix arrays, we assess the performance of the Human Exon 1.0 ST (HuEx) and U133 Plus 2.0 (U133Plus2) platforms directly through a series of ‘spike-in’ hybridizations containing 25 transcripts in the presence of a fixed eukaryotic background. Specifically, we compare the measures of expression for HuEx and U133Plus2 arrays to evaluate the precision of these measures as well as the specificity and sensitivity of the measures' ability to detect differential expression.

**Significance:**

This study presents an experimental comparison and systematic cross-validation of Affymetrix exon arrays and establishes high comparability of expression changes and probe performance characteristics between Affymetrix conventional and exon arrays. In addition, this study offers a reliable benchmark data set for the comparison of competing exon expression measures, the selection of methods suitable for mapping exon array measures to the wealth of previously generated microarray data, as well as the development of more advanced methods for exon- and transcript-level expression summarization.

## Introduction

We are witnessing a rapid evolution of microarray technology with the potential for clinical application in diagnostics, therapeutic target identification, patient risk stratification, and pre-clinical toxicology and drug development. Today, Affymetrix GeneChip Exon Array system provides the opportunity to interrogate over one million RNA transcripts on a single array [1].

The oligonucleotide probes of exon arrays radically differ from those on conventional 3′ expression arrays in their design, density, and coverage. While the conventional Affymetrix GeneChips (e.g. U133Plus2) feature a probeset consisting of 11–20 probes selected from the 3′ end of the mRNA sequence, the new all-exon arrays (e.g. Human Exon 1.0 ST), in contrast, have a mere 4 probes selected from each putative exonic region (see Affymetrix technical documentation for Exon Array probe annotations). To generate the target, HuEx arrays use T7 linked random hexamers for cDNA synthesis as opposed to all previous Affymetrix expression arrays, which employ an oligo-dT linked T7 and thus require an intact poly-A tail. Importantly, this new WT Sense Target Labeling Assay generates DNA targets and therefore results in DNA/DNA duplex formation during hybridization, as opposed to DNA/RNA heteroduplexes in conventional arrays. While first attempts to describe the performance of HuEx in comparison to conventional arrays have been made [2], systematic studies of signal behavior on HuEx arrays have not been described, and comparative analysis of this behavior with 3′ targeted arrays like the U133Plus2 series is unknown. A number of physical models have been proposed to describe DNA/RNA hybridization on expression arrays in the past [3], [4], but none for DNA/DNA interactions as seen on the HuEx arrays. Affymetrix Latin-square spike-in datasets were pivotal in the development of these realistic physical models, reliable analysis algorithms, and the establishment of benchmarks for conventional GeneChip expression measures. However, no similar studies have been published for the new exon arrays to study the DNA/DNA hybridization mechanism. By offering a missing experimental component, our study was designed to evaluate the sensitivity of HuEx signal and the platform's ability to detect differential expression in a wide range of target concentrations as well as to compare all-exon array performance to conventional U133Plus2 arrays.

This controlled experiment was designed to follow a Latin Square with 25 Human clones arranged in 5 spike-in gene groups at 5 concentrations in the presence of a complex background, repeated in triplicate. Additionally, we used the same experimental design and same samples to generate measures of expression for Affymetrix U133Plus2, thereby allowing an unbiased comparison of the two platforms. This study offers a reliable benchmark data set that may be used for the comparison of 1) competing exon expression measures, 2) the variety of methods suitable for mapping exon array measures to the wealth of previously generated microarray data as well as 3) the development of more advanced exon- and transcript-level expression summarization methods.

## Results

### Platform Representation of the Selected Clones

To establish platform performance and cross-platform comparisons, a set of 25 sequence-verified spike-in mRNAs was hybridized to both U133Plus2 and HuEx arrays according to a Latin Square experimental design as described in Materials and Methods. Affymetrix probeset annotations were used to establish that these 25 spike-in clones are represented by a total of 112 probesets on U133Plus2 and 1075 probesets on HuEx. It should be noted that while U133Plus2 arrays have an average of one probeset per transcript, our clone selection process was designed to provide an over-representation of probesets on U133Plus2 arrays that would allow addressing possible alternative polyadenylation sites and possible alternative 3′ transcript ends. Due to an oversight in clone selection, one of the clones, I.M.A.G.E. 4843460, was not presented in either U133Plus2 or HuEx arrays, i.e. none of the probes were found to be complementary to the clone sequence. Thus, we exclude this clone from further analysis of probe signal. See Table 1 for a detailed summary of clone representation.

**Table 1:** Summary of Spike-in Clones Representation in Affymetrix Platforms

U133Plus2					HuEx 1.0			
	Probesets	Probes	Probes Present	Probes Working	Probesets	Probes	Probes Present	Probes Working
AAK1	8	88	11	11	110	429	49	45
ARL6IP2	6	66	4	4	58	229	48	40
C1orf187	3	33	11	11	18	61	24	23
COPS4	2	22	11	11	28	99	46	46
EDNRB	3	33	21	21	55	206	47	47
GALK2	9	99	14	13	59	228	50	49
GFRA1	5	60	0	105	408	41	39	
GLYATL1	3	33	11	11	78	290	46	45
INHBA	2	22	11	11	19	76	34	34
KCNH6	3	33	22	22	32	119	37	35
KRT7	4	44	11	11	32	123	54	16
MGC10646	3	33	11	11	15	57	16	16
MRPS5	5	55	22	22	31	122	62	59
MRS2L	6	66	11	6	36	138	42	42
NOSTRIN	2	22	11	11	48	184	70	69
PAX9	2	22	10	10	20	77	21	21
POU2F2	9	99	11	11	50	186	72	71
RPIP8	3	33	11	11	28	107	48	48
SEC22B	6	66	11	6	11	42	12	5
SERGEF	6	66	33	33	57	213	65	61
SLC39A14	4	44	22	22	33	126	43	41
SNTB2	10	110	11	11	41	159	48	46
SNX24	6	66	11	11	62	245	31	31
TRIM55	2	22	4	4	49	189	55	54
**Total**	**112**	**1237**	**306**	**295**	**1075**	**4113**	**1061**	**983**

### Signal Response to Concentration and Isotherm Fit

The majority of both U133Plus2 and HuEx probes that are complementary to spike-in targets respond to concentration changes in a non-linear Langmuir fashion, as previously reported for U95 and U133A arrays [3]. 78 (or 7.3%) of HuEx 1.0 and 11 (or 3.6%) U133Plus2 probes with sequence complementary to target did not respond to changes in concentrations; see Supporting Tables S1 and S2 for probe summaries. These probes were excluded from further analysis.

**Figure 1:**
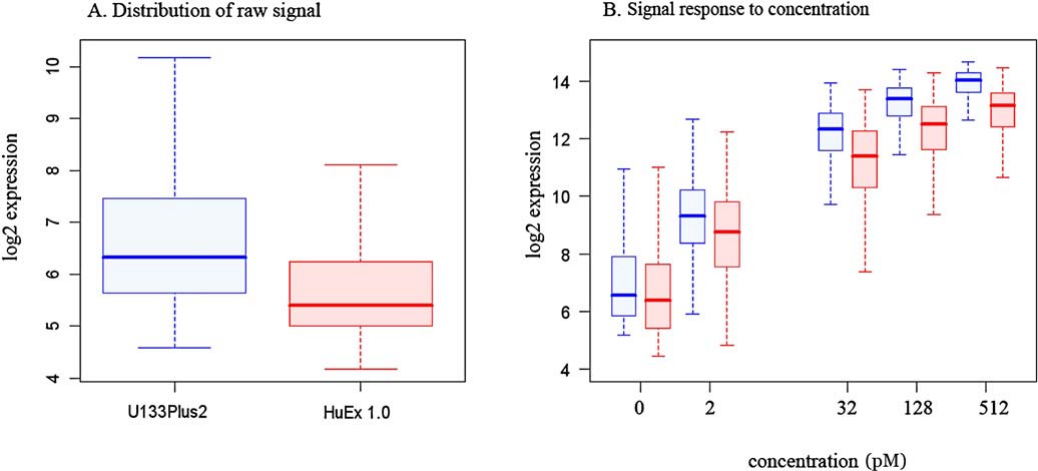
A) Distribution of array intensities for all probesets in HuEx 1.0 [red] and U133Plus2 [blue]. B) Signal response to spike-in concentrations in HuEx 1.0 [red] and U133Plus2 [blue].

Next we examined the overall probe signal behavior on both platforms. Figure 1, Panel A shows a side-by-side distribution of log2 raw signal intensities with medians of 5.4 and 6.3 for HuEx and U133Plus2 arrays, respectively. The observed decrease in median fluorescent intensity of HuEx arrays can be explained by differences in information content, i.e. number of probes and probesets represented by each platform. HuEx 1.0 arrays were designed to represent annotated transcripts from primary sequence databases and gene prediction sets as well as many unannotated transcripts of unknown function, antisense and intronic probesets. Therefore HuEx contains a higher proportion of targets that are expected to be less frequently or not expressed. Additionally, changes in probe nucleotides composition as well as differences in DNA/DNA vs. DNA/RNA binding properties [5], [6] may alter mismatch discrimination abilities of the platforms and contribute to differences in dynamic range. In addition to reduced signal intensity, we also observe reduced dynamic range of HuEx signal, see Figure 1 Panel.

**Figure 2:**
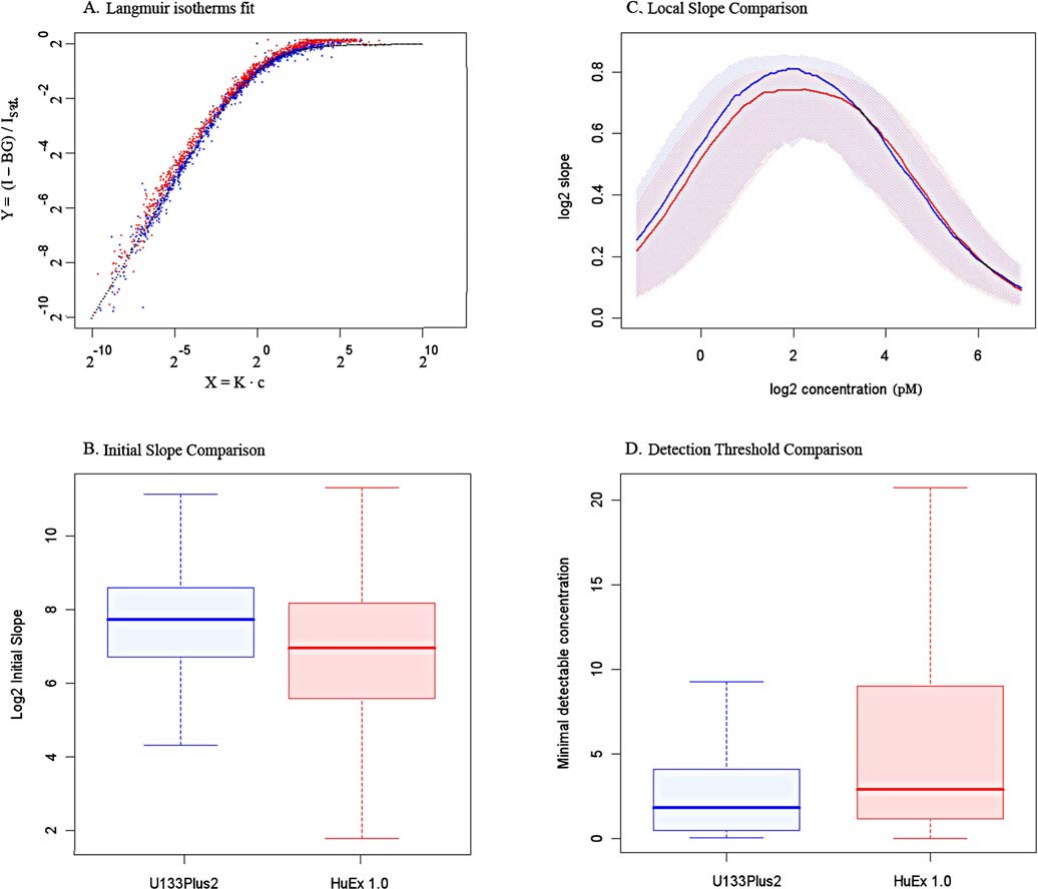
A) Model fit for HuEx [red] and U133Plus2 [blue]. After each probe has been fit to the model, described in Material and Methods, the rescaled variables 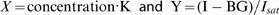 overlay the classical Langmuir adsorption isotherm, shown in dotted black. B) Distribution of Initial Slopes for HuEx [red boxplot] and U133Plus2 [blue boxplot] spiked probes. C) Local slopes as a function of concentration for HuEx [median red line and inter-quartile range shaded red] vs U133Plus2 [median blue line and inter-quartile range shaded blue]. Local slopes represent the fitted log2 fold-change for probes with true log2 fold-change of 1 as a function of the nominal concentration. D) Distribution of detection threshold for HuEx 1.0 [red boxplot] and U133Plus2 [blue boxplot] spiked probes. Detection threshold is referred to probe concentration where signal-to-noise ratio exceeds 3.

Examining individual probe signal response curves, we observe that HuEx 1.0 raw intensity signal follows a hyperbolic function, similar to the one observed in conventional Affymetrix arrays. We fit a non-linear Langmuir-inspired model, introduced in the Methods section, to raw signal for both platforms and extract the probe parameters. As shown in Figure 2, Panel A a transformation of raw signal, using parameters, obtained in the fit, collapses the data tightly onto theoretical prediction, suggesting that the Langmuir model thoroughly captures the physical chemistry of GeneChip hybridization on both U133Plus2 and HuEx 1.0 platforms.

### HuEx 1.0 vs U133Plus2 Cross-platforms Comparison

Due to saturation, the slope of non-linear signal response to concentration decreases with the increase in spike-in concentration. The maximum sensitivity should then theoretically be observed at small concentrations, providing initial slope as a measure of absolute probe responsiveness. Based on fitted probe parameters, we calculate initial slope for all probes across both platforms at 1 pM and observe that median log2 initial slope in HuEx is 7.0 and is 1.7 times smaller on natural scale than in U133Plus2 with median log2 initial slope equal to 7.7, see Figure 2, Panel B.

Even though we found that the average responsiveness of HuEx 1.0 probes is lesser than U133Plus2 probes i.e. the same change in transcript concentration leads to smaller absolute intensity increase, this characteristic is less important for traditional relative comparison of two or more conditions where the ratio of intensities is calculated. A more informative measure of performance in such comparisons is a log-log slope metric-a slope of signal response to concentration on a log scale that indicates the rate of change, i.e. how much log signal increases in response to a unit of concentration change in different concentration ranges.

Comparison of log-log slopes showed that U133Plus2 is more sensitive, with median slope attaining a maximum of 0.86 in U133Plus2 vs 0.78 in HuEx 1.0, though the difference between platforms in regards to local slopes is significantly less than in absolute probes affinity comparisons; see Figure 2, Panel C.

The detection sensitivity of both platforms was assessed by measuring the smallest probe concentration at which signal-to-background ratio exceeds 3. We found that median probe detection threshold is 1.84 pM for U133Plus2 and 2.92 pM for HuEx 1.0; see Figure 2, Panel D for detection threshold distributions for both platforms.

### Transcript-Level Summary and Cross-Platform Comparison

**Figure 3:**
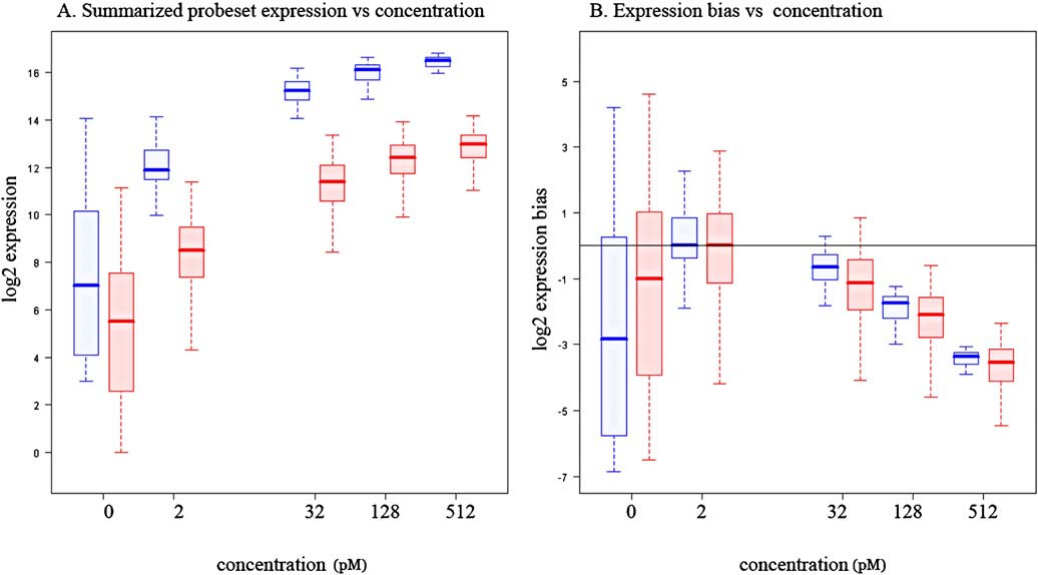
A) Summarized expression measures response to log2 concentration in HuEx 1.0 [red] and U133Plus2 [blue]. B) Expression bias as a function of log2 concentration in HuEx 1.0 [red] and U133Plus2 [blue].

Because the number of competing summarization methods is large and growing, a thorough and systematic assessment of their performance deserves an independent effort and is outside of the scope of this study. While avoiding comparisons of summarization methods, we focus on probeset-level summaries for both platforms, obtained using Affymetrix Expression Console tools, described in Material and Methods. Summarized probeset signal response to nominal concentration is shown on Figure 3 where Panel A plots log2 summarized expression measures vs. log2 of nominal concentration and Panel B represents the relative bias in concentration detection, i.e. the difference between summarized expression measures and nominal concentration shifted arbitrarily so that the zero value is at 2 pM spike. While an apparent difference in probeset expression measures in different array platforms is observed on Panel A, the ability to detect difference in fold changes for both platforms is comparable as seen in Panel B.

## Discussion

This study presents a comparison of two Affymetrix platforms–market leading U133Plus2 and relatively new all-exon HuEx arrays, which provide for the first time exon-level expression profiling at the whole-genome scale. Despite several major technological changes, we observe a high concordance between these platforms; i.e. individual HuEx probes are capable to reliably detect concentration changes and thus provide unbiased expression measures at exon level. We showed that Langmuir isotherms, devised earlier for previous generations of Affymetrix GeneChips, accurately capture the non-linear relation between measured intensity and concentration for both U133Plus2 and HuEx 1.0 arrays. Using a non-linear physical model, we estimated individual probe parameters and proceeded to compare platform performance in terms of signal sensitivity and specificity. We found that probe intensity changes in response to concentration in HuEx arrays exhibit a smaller dynamic range, higher detection threshold, and decreased sensitivity compared to U133Plus2.

A critical issue for Affymetrix GeneChip™ users is HuEx compatibility to previous generations of Affymetrix arrays. Vast amounts of U133plus2 and earlier data are publicly available, but their comparability with new GeneChip arrays like the HuEx is unknown. Evidence that data from both sources can be reliably compared is of critical importance. The results reported here are thus reassuring. As we showed in log-log slope analysis, median relative sensitivity is similar in both platforms, suggesting that an accurate cross-platform mapping can be established. However, differences in detection thresholds demand special attention to this problem. While offering a basic summarized signal comparison, we recognize the need for further improvements in summarization routines for all-exon arrays. By releasing the results of this study to the academic community, we hope to facilitate the comparison of competing exon- and transcript-level expression measures, alternative splicing detection, and selection of methods suitable for mapping exon array measures to the wealth of previously generated microarray data.

## Materials and Methods

### Clone Selection

Using publicly available microarray data, deposited in NCBIs Gene Expression Omnibus (GEO, http://www.ncbi.nlm.nih.gov/geo/) and accessible through GEO Series accession number GSE5823, DNA clones for this experiment were selected based on their lack of expression in total RNA isolated from HeLa cells. The only other restrictions on clone selection were 1) the presence of both a polyA tail and a XhoI restriction site at the 3′ end of the coding sequence to simplify linearization of cDNA and 2) a size of 1400 to 1800 bases. Due to alternative polyadenylation sites and variant transcripts that may be differently regulated, some genes are represented by multiple probesets in the design of U133Plus2 arrays. In order to address this redundancy and to compare the sensitivity of these probesets, we specifically selected clones that were represented by more than one probeset in U133Plus2 arrays. Clone information is summarized in supplementary materials; see Supporting Table S3.

### Target Preparation

cDNA clones with ORFs ranging from 1,400 to 1,800 nucleotides were purchased from Open Biosystems (Open Biosystems, Huntsville, AL, USA) as glycerol stocks in the pOTB7 vector. Each cDNA was bacterially amplified, isolated, and sequenced with vector specific primers (M13F and M13R). Following linearization with Xho1, each cDNA was column purified (Qiagen) and *in vitro* transcribed into mRNA (AmpliScribe(tm) SP6-Flash Transcription Kit). The resulting mRNA was then purified (RNeasy columns-Qiagen), quantified (Nanodrop), and analyzed (Nanochips and Bioanalyzer 2100–Agilent) to ensure full length mRNA products. The 25 full-length mRNAs that passed our requirements were serially diluted and then pooled into 5 ‘spike-in’ RNA mixes. These mixes (<3 ng total) were added to HeLa cell total RNA (10 ug) and aliquots of the same RNA mix were used for carrying out both the WT Sense Target Labeling Assay for HuEx arrays and One-Cycle Target Labeling Assay for U133Plus 2 arrays.

### Experimental Design

Human cRNA transcripts were spiked into labeled complex human backgrounds at known concentrations, and hybridization intensities were obtained for HuEx and U133Plus2 arrays. Target groups were arranged in a classic Latin square design so that each hybridization mixture contained five targets at each chosen target concentration. 25 cRNA targets were spiked at 5 concentrations that included 0, 2, 32, 128, and 512 pM; see Supporting Table S3 for a list of clones. Thus, this data set consists of 3 technical replicates of 5 separate hybridizations of 25 spiked transcripts in a complex human background at concentrations ranging from 0 pM to 512 pM. Hybridization and wash conditions were the same as indicated in the Affymetrix gene expression manuals. Hybridization intensities were generated for the experiments according to the standard procedures for GeneChip expression probe arrays.

### Target Hybridization and Data Collection

Arrays were washed and stained according to standard Affymetrix protocols using an Affymetrix Fluidics Workstation. Arrays were scanned using an Affymetrix GeneArray scanner and the cell intensities (.CEL files) were captured by the Affymetrix GeneChip Operating Software (GCOS). To allow for direct comparison of probe intensities across multiple arrays within the same platform we applied a locally weighted linear regression, implemented as R routine lowess() [7], independently to each platform in order to normalize raw probe intensities.

### Model-Based Analysis of Probe Response

The main operational principal of microarrays is based on specific binding of DNA or RNA target to oligonucleotide probes synthesized on the array surface. Previous generations of conventional Affymetrix microarrays (e.g. U133Plus2) relied on the formation of RNA/DNA hetero-duplexes where attached DNA probes featured exact complementary sequences to the target RNA. In contrast, new HuEx arrays rely on more selective DNA/DNA binding with significantly different physical properties. Additionally, the HuEx platform pioneers a new data structure where individual probesets represent distinct exon or RNA transcript expression that can be assembled into global transcript expression measures. These changes in array design and sample preparation protocols present significant challenges in the application of existing analysis methods. One of the main goals of this study is to access and quantify the differences and similarities between conventional and all-exon Affymetrix platforms.

In recent years, DNA/RNA duplex formation in microarrays has been studied extensively. In particular, it was shown [3], [6], [8] that the nonlinear response of microarray hybridization intensity to the transcript concentration is best described by the hyperbolic function, known as the Langmuir adsorption isotherm:
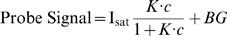
where *I_sat_* is a maximum attainable probe intensity, *K* is an equilibrium constant, *c* is target concentration and *BG* is the background component of the signal, i.e. probe signal in the absence of target transcript that represents probe response to complex genomic background. Nonlinearity, caused by saturation effects, varies significantly for different probes depending on the values of the equilibrium constant *K*. To assess platform performance, we study and compare signal dynamic range, minimal detectable concentration and responsiveness of probes to concentration changes.

We assess individual probe performance by fitting the Langmuir isotherm to the hybridization intensity of spiked probes vs known spike concentration. We adapt a non-linear fitting routine [9] that uses a least square nlm() optimization routine in R. Briefly, multimodel inference, incorporated within the NLFIT fitting routine [9], involves a full model above, where *c* varies across arrays as well as reduced model, where *c* is constant across all conditions. Evaluating model goodness-of-fit, i.e., measuring quantitatively the agreement between a proposed model and the data, allows to observe that responding probes result in significantly reduced residuals sum square for the full nonlinear model compared to the null model, while probes that do not respond to concentration changes do not benefit from extending the model. Hence, for each probe in U133Plus2 and HuEx we calculate *R^2^* coefficients *(RSS_0_-RSS_fit_)/RSS_o_*, where *RSS_0_* denotes the residual sum of squares from fitting the reduced model and *RSS_fit_* denotes the residual sum of squares from fitting the full model. Probes that demonstrate *R^2^* values above a 0.95 cutoff are considered to be responsive and selected for further performance analysis.

### Log-Log Slope Calculation

Log-log slopes represent the observed log fold-change for probes with true fold-change of 1 as a function of the total nominal concentration. In the case of Affymetrix Latin Square experiments, where each consecutive experimental concentration doubles in the [1..512 pM] range, local slope is simply the difference between log signal in consecutive experiment. In this study, a reduced Latin Square design was used, resulting in irregular concentration intervals with observation over only 5 concentrations. Thus, a numeric estimation will not be accurate and we resort to estimating log-log slope based on the signal model introduced above, where
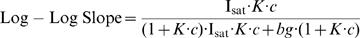



### Detection Threshold Calculation

Clearly, the absolute level of background noise will affect the detection threshold of the array platform. The spatial variation of the signal due to random non-specific hybridization reduces sensitivity by adding independent noise and compromises our ability to detect differential expression changes in low concentration ranges. Signal-to-noise (SNR) ratio is used in many signal-detection disciplines as a quantitative measure of the ability to resolve true signal from background noise. In microarrays, SNR quantifies how well specific signal is resolved from the non-specific noise. SNR is commonly calculated as: *(Signal-Background)/SD(background)*. A higher SNR indicates higher signal over background noise; a signal-to-noise ratio of 3 is commonly considered the lower limit for accurate detection. Signal may be detected below this value, but the accuracy of quantitative measurements decreases significantly.

In low concentration area, where saturation effects are negligible, the signal can be represented by *I = I_sat_·K·c* and SNR can be then defined as: 
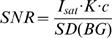



The derivation of the detection threshold requires a priori knowledge of the mean and variance of background. Latin square design, used in this study, allows us to estimate the non-specific background from the zero-spike concentration, where target is not present in the mix. We obtain SNR for each probe in both platforms and define detection threshold as concentration where SNR exceeds 3.

### Transcript Level Summarization

We summarized transcript level for U133Plus2 and HuEx 1.0 using standard analysis tools, provided by Affymetrix. For U133Plus2 arrays, we ran Probe Logarithmic Intensity Error Estimation (PLIER) within Affymetrix Expression Console software. For HuEx arrays, Affymetrix Exon Array Computational Tool (ExACT) software package was used to generate PLIER-summarized probeset expressions.

## Supporting Information

Table S1 Human Exon Array Spike-In Probes Summary. We assess individual probe performance by fitting the Langmuir isotherm to the hybridization intensity of spiked probes vs known spike concentration. Human Exon Array probes that demonstrate R2 values above a 0.95 cutoff are considered to be responsive and summarized in this table.
(0.46 MB XLS)

Table S2 U133Plus2 Array Spike-In Probes Summary. We assess individual probe performance by fitting the Langmuir isotherm to the hybridization intensity of spiked probes vs known spike concentration. U133Plus2 Array probes that demonstrate R2 values above a 0.95 cutoff are considered to be responsive and summarized in this table.
(0.09 MB XLS)

Table S3 Spike-In Clone Summary.
(0.02 MB XLS)
